# Canibacter oris – a fairly unknown pathogenic agent of bite wound infections

**DOI:** 10.3205/id000070

**Published:** 2021-04-22

**Authors:** Herbert Hof, Konrad Bode, Falko von Stillfried

**Affiliations:** 1 MVZ Dr. Limbach und Kollegen, Heidelberg, Germany; 2 Sankt Vincentius Krankenhaus, Klinik für Rekonstruktive und Plastische Chirurgie, Handchirurgie, Speyer, Germany

**Keywords:** Canibacter oris, class of Actinobacterium, PCR, MALDI-TOF, bite wound infection

## Abstract

Here, we report on the second case of bite wound infection by *Canibacter oris*. This bacterium belongs to the family of *Microbacteriaceae* in the order of *Microbacterales* in the class of *Actinobacteria*, which are prevalent in the oral flora. Possibly this bacterium has been overlooked until now, because it cannot be recognized by conventional differentiation methods. MALDI-TOF as well as PCR are able to identify this pathogen.

## Introduction

Up to 50,000 bite wounds caused primarily by dogs and less often by cats, humans, and small animals like rats occur in Germany every year. Predominantly children are affected, whereby the hands are exposed most. The risk of manifest infection after a bite injury is about 10% due to pathogens residing in the oral microbiome of animals and humans, respectively [1], [2]. A typical agent of dog and cat bite wounds is *Pasteurella multocida* [3], whereas *Streptobacillus moniliformis* is typically found in rat bite wounds [4]. In addition to these key bacteria, several others, such as *Staphylococcus aureus*, *Streptococcus pyogenes*, *Bacteroides* spp., *Fusobacterium* spp., *Eikenella corrodens*, *Capnocytophaga* spp. and *Aggregibacter* spp. are found relatively often [4]. But obviously much more fastidious bacteria like *Actinobacteria* are present in animal-caused bite wounds when special culture conditions and molecular methods for differentiation are used [5]. So far, *Canibacter oris*, which belongs to this class of bacteria, has only been isolated once before from a human wound in Australia caused by a dog bite [6].

Here, we report on a second human case of wound infection after a dog bite caused by *C. oris*.

## Case description

A 46-year-old woman tried to separate her dog from another, unknown dog that had attacked her dog. In the process, she was bitten into her left hand by the other dog. As soon as within 2 days, a purulent, phlegmonous inflammation developed at the site of the bite wound in spite of a calculated antibiotic therapy with ampicillin + sulbactam (3x3 g iv/day). The patient had no systemic signs of infection, i.e. no fever (37.2°C) or malaise. Furthermore, the blood leukocyte count was normal (8.90x10^3^/µl) and C-reactive protein (CRP) was within normal range (0.35 mg/dl).

A surgical debridement accompanied by a synovialectomy of the extensor tendons and the carpo-metacarpal joint of the thumb was done. The purulent material obtained during the operative intervention was sent to the laboratory for microbiological examination. A lot of colonies of *Canibacter oris* were isolated 24 hours later, as well as *Pasteurella multocida* and some further aerobic and anaerobic bacteria.

After the operation, the patient recovered under continued ampicillin + sulbactan regimen (3x3 g iv/day), yet slowly. After 5 days, since the wound had become dry and non-irritant, the patient could be discharged from the hospital. There was no follow-up afterwards.

## Microbiology

One day after incubation at 36°C, six different colonies were isolated on sheep blood agar plates (Oxoid, Wesel) incubated aerobically or anaerobically, respectively. These colonies were identified by MALDI-TOF MS (Bruker, Bremen) as *Pasteurella multocida* (abundant), *C. oris *(moderate), *Streptococus minor* (moderate), *Actimomyces canis* (moderate) and two different *Prevotella* spp. (rare).

*C. oris* grown under aerobic conditions could not be identified by biochemical methods (VITEK, Biomérieux, Nürtingen), whereas the final identification could be confirmed by PCR, i.e. by 16S rDNA Sequencing Test (Micro-DxTM PC; Molzym Bremen) Dan Quick BioInformatic Phylogeny of Prokaryotes – QBPP v 1.1.

**Figure 1:**
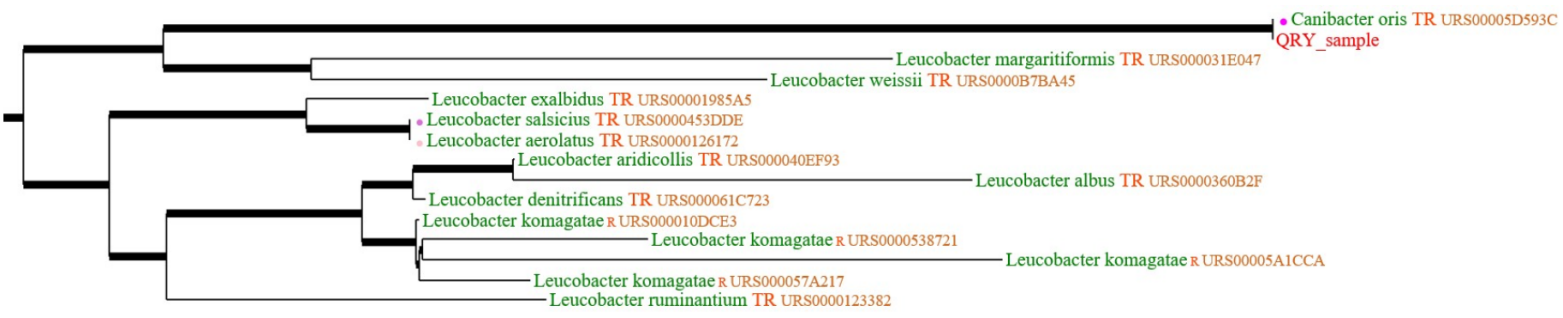
Dendrogramme of the genetic relationship of the isolate of Canibacter oris with the other Canibacter oris strain and with Leucobacter spp.; Quick BioInformatic Phylogeny of Prokaryotes (https://umr5558-bibiserv.univ-lyon1.fr/lebibi/lebibi.cgi)

The nucleoidacid sequence of the16S rRNA gene of the isolate revealed that it was identical with that of the first described *C. oris* strain isolated so far [7], and quite closely related to *Leucobacter* spp., which belongs to the order of *Micrococcales*, and to the family of *Microbacteriaceae* in particular (Figure 1 (Fig. 1)).

An antibiogram was only obtained from *P. multocida*, since this was the most abundant bacterium. A typical pattern was observed showing susceptibility to penicillin and ampicillin. Unfortunately, the antibiotic susceptibility of the *C. oris* isolate has not been determined.

## Discussion

The composition of the canine oral microbiome is quite complex. *Firmicutes*, *Proteobacteria*, *Bacteroidetes* and *Actinobacteria* are the most prevalent bacteria [7], [8], [9]. Human pathogenic bacteria are among them as well. Indeed, in many cases of bite wound infections, polymicrobial constellations are found. Furthermore, one has to be aware of complications due to unusual bacteria [2], resulting not only in localized infections, but also in multiple metastases in various organs because of systemic spreading [3].

*C. oris* is a grampositive rod [6] which has been described in detail by Trujillo [10], who classified this bacterium into the class of non-motile *Actinobacteria*. It belongs to the order of *Microbacterales* and the family of *Microbacteriaceae*, which are quite closely related to *Leucobacter* spp. (Figure 1 (Fig. 1)). As Figure 1 (Fig. 1) shows, *Actinobacteriae* are prevalent in the oral microbiome of dogs [7], [8], [9].

The pathogenic role of *C. oris* is not well understood, and virulence factors in particular have not been described yet. However, it can be assumed that *C. oris* possesses certain pathogenic properties. As previously described, this bacterium was the only pathogen in a bite wound and has been considered responsible for the inflammatory reaction of the infected tissue [6]. Presumably, this pathogenic bacterium, along with other well-known pathogens such as *Pasteurella multocida*, will have contributed to the development of the purulent inflammation seen in our patient. Indeed, bite wound infections are often caused by a combination of several pathogens acting synergistically [3], whereby it remains generally unclear which respective role can be attributed to each of the several bacteria present in such a polymicrobial infection.

Albeit *C. oris* is rarely isolated from bite wounds, it can be assumed that this bacterium might be a much more frequent agent than has been described in the literature until today. The following items might explain why *C. oris* has not been found more frequently in bite wounds so far.

- In many cases of bite wounds, no microbiological examination is ordered at all.
- It can be doubted whether all bacteria present in a bite wound will be routinely detected by culture.
- In many cases, several bacteria are present that may overgrow *C. oris*, because this bacterium is microaerophilic, which represents a relative disadvantage in routine culture conditions.
- Potentially, the common bacteria present in bite wounds are dominant and *C. oris* is overlooked, since not all of the isolates will be differentiated in routine situations because of economic reasons.
- Misidentification might occur, since the classical method of identification of bacteria by means of biochemical methods will fail, because the databases of some commercial test systems do not contain this rare code yet.
- Possibly, this bacterium has been found but this result was not communicated.

It remains unclear why a manifest inflammation develops in only 10% of animal bites. Although a microbiological examination is not necessary in those cases with no clinical signs, it should be deliberated whether an antibiotic prophylaxis should be given in addition to antiseptic dressing and immobilization. This holds especially true in the case of hand bites, deep lesions (typically induced by cats) and crushing, since the complications and the sequelae of such infections could be severe [3].

In case of inflammatory reactions, a microbiological examination is recommended since the causative pathogens can vary. Antibiotic susceptibility testing of the isolates should be done in general as well, so that a directed antibiotic therapy could be given to avoid large inflammatory infiltrations, tissue destructions and even dissemination. The antibiotic susceptibilities of *C. oris* remain unclear since unfortunately no antibiogram was done from the present isolate, and since in the previous descriptions of the first isolate of *C. oris* [6], [10] information about its susceptibility to antibiotics is lacking.

Since anaerobes like *Prevotella* spp. and *Clostridium* spp. that often produce betalactamases are rather frequently found among the complex bacterial flora in bite wounds [4], it is advisable to protect ampicillin by betalactamase inhibitors such as sulbactam. Since most antibiotics do not penetrate well into necrotic and purulent tissues, such antibiotic regimens are only indicated in addition to surgical sanitation.

## Notes

### Competing interests

The authors declare that they have no competing interests.
